# First Evidence of Amphibian Chytrid Fungus (*Batrachochytrium dendrobatidis*) and Ranavirus in Hong Kong Amphibian Trade

**DOI:** 10.1371/journal.pone.0090750

**Published:** 2014-03-05

**Authors:** Jonathan E. Kolby, Kristine M. Smith, Lee Berger, William B Karesh, Asa Preston, Allan P. Pessier, Lee F. Skerratt

**Affiliations:** 1 One Health Research Group, School of Public Health, Tropical Medicine, and Rehabilitation Sciences and One Health Research Group, James Cook University, Townsville, Queensland, Australia; 2 EcoHealth Alliance, New York, New York, United States of America; 3 Amphibian Disease Laboratory, Institute for Conservation Research, San Diego Zoo Global, San Diego, California, United States of America; Imperial College, United Kingdom

## Abstract

The emerging infectious amphibian diseases caused by amphibian chytrid fungus (*Batrachochytrium dendrobatidis*, *Bd*) and ranaviruses are responsible for global amphibian population declines and extinctions. Although likely to have been spread by a variety of activities, transcontinental dispersal appears closely associated with the international trade in live amphibians. The territory of Hong Kong reports frequent, high volume trade in amphibians, and yet the presence of *Bd* and ranavirus have not previously been detected in either traded or free-ranging amphibians. In 2012, a prospective surveillance project was conducted to investigate the presence of these pathogens in commercial shipments of live amphibians exported from Hong Kong International Airport. Analysis of skin (*Bd*) and cloacal (ranavirus) swabs by quantitative PCR detected pathogen presence in 31/265 (11.7%) and in 105/185 (56.8%) of amphibians, respectively. In addition, the water in which animals were transported tested positive for *Bd*, demonstrating the risk of pathogen pollution by the disposal of untreated wastewater. It is uncertain whether *Bd* and ranavirus remain contained within Hong Kong’s trade sector, or if native amphibians have already been exposed. Rapid response efforts are now urgently needed to determine current pathogen distribution in Hong Kong, evaluate potential trade-associated exposure to free-ranging amphibians, and identify opportunities to prevent disease establishment.

## Introduction

The high volume of global trade in potentially diseased amphibians has sparked a series of investigations into its role as a primary driver of the emergence and spread of amphibian chytrid fungus (*Batrachochytrium dendrobatidis*, *Bd*) and ranaviruses, threatening global amphibian biodiversity [1]–[5]. With respect to *Bd*, particular concern has been expressed regarding the transport of American bullfrogs (*Lithobates catesbeianus*), due to the species' propensity to carry infection asymptomatically and serve as a reservoir of disease [6], [7]. Millions of *L. catesbeianus* are traded globally for consumption annually. High prevalence of *Bd* infection (41–62%) has been detected among this species sold in markets in the USA, imported primarily from Southeast Asia and South America [1]–[2]. Furthermore, *Bd*-positive water often accompanying commercial amphibian shipments likewise represent a potential source of spread [8]–[9].

Similarly, ranaviruses are emerging pathogens capable of causing mass mortality and localized population decline in amphibians [10], as well as reptiles and fish, and their spread shares many nuances with the global dispersal of *Bd* [11]. Transmission of viral particles occurs through direct contact with infected individuals and exposure to contaminated water or soil. Its ability to infect three classes of ectotherms and the lack of an effective therapeutic treatment warrants serious consideration. The geographic spread of ranavirus also demonstrates strong association with the trade in live amphibians, most notably the trade in tiger salamanders (*Ambystoma tigrinum*) and American bullfrogs [2], [12].

Investigations for the presence of these pathogens in both traded and free-ranging amphibians in Asian countries have produced mixed results, ranging from lack of detection to widespread low prevalence [5], [13]–[16]. Previous surveillance efforts have not detected *Bd* in Hong Kong, a global amphibian trade hub, despite substantive testing of bullfrogs imported for consumption, native free-ranging amphibians, and non-native pet species [16, Simon Chan pers. comm. 2012). Furthermore, neither amphibian mass mortality events nor enigmatic population declines have been documented in Hong Kong suggesting pathogen absence. However, pre-metamorphic and recently metamorphosed amphibians are often more susceptible to *Bd* and ranavirus [17]–[20], and previous surveys have only concentrated on adults, potentially reducing survey sensitivity. Surveys of imported bullfrogs (*L. catesbeianus*) sold for consumption in the USA have demonstrated a high prevalence of infection with ranavirus and *Bd* suggesting a foreign source [2], [3]. Therefore, recognizing the current risk of pathogen pollution from regional trading partners with known presence of these pathogens, the aim of this study was to identify the potential presence of *Bd* and ranavirus in Hong Kong trade by examining amphibians commercially exported to the USA.

## Methods

### Ethics

This study was approved by Tufts University’s Institutional Animal Care and Use Committee (Permit #G2010-85). Amphibians were sampled upon importation with permission from the United States Fish and Wildlife Service.

### Amphibian trade data

A request was made to the United States Fish and Wildlife Service (USFWS) for all records describing the country's trade in amphibians from 1 January 2006 to 11 October 2011. Information characterizing the international trade in wildlife is maintained in the USFWS Law Enforcement Management Information Systems (LEMIS) and made available through the Freedom of Information Act (FOIA). Records provided by USFWS were filtered to include only specimens of live amphibians commercially exported by Hong Kong to the USA and subtotaled by species. These data were used to evaluate the potential for amphibian pathogen presence in traded species and identify which of those species were most likely to be available for sampling.

### Sample Collection

Amphibians were sampled in the USA immediately upon arrival from Hong Kong International Airport between May and September 2012. Permission to sample amphibians was provided by the USFWS. Four amphibian species commonly traded in high volumes were targeted for sampling (Table 1), including those with known or expected pathogen susceptibility, to increase the likelihood of pathogen detection in each shipment. These species are typically maintained and shipped in an aqueous environment and in high densities, providing conditions likely to increase pathogen transmission for both *Bd* and ranavirus. The importation documents presented with each shipment declared that all specimens had been bred in captivity in Hong Kong. A record was made of the importation date, the total number of specimens present, substrate type, and physical condition of each sampled amphibian.

**Table 1:** Summary of live amphibians imported into the USA from Hong Kong during a 5-year period (1 January 2006 -26 December 2010).

Species	Quantity
*Hymenochirus curtipes*	1468130
Xenopus laevis*	673859
Cynops orientalis*	374560
Triturus hongkongensis*	216054
*Hymenochirus boulengeri*	207632
Bombina orientalis*	190189
*Hymenochirus boettgeri*	102160
*Cynops pyrrhogaster*	83178
*Xenopus sp.*	82996
*Triturus sp.*	59065
*Pachytriton brevipes*	42613
*Cynops sp.*	27703
*Non-CITES amphibian species*	19027
Paramesotriton hongkongensis*	17870
*Hymenochirus sp.*	8870
*Physalaemus sp.*	7200
*Eleutherodactylus sp.*	4500
*Pachytriton labiatus*	3584
*Hyla sp.*	2104
*Tylototriton kweichowensis*	1501
*Paramesotriton chinensis*	1491
*Pachytriton sp.*	1172
*Tylototriton verrucosus*	1160
*Tylototriton sp.*	939
*Polypedates dennysii*	903
*Rhacophorus sp.*	524
*Xenopus clivii*	400
*Pachyhynobius shangchengensis*	343
*Silurana sp.*	300
*Bombina bombina*	290
*Polypedates sp.*	236
*Rhacophorus dennysi*	226
*Tylototriton shanjing*	190
*Hyla arborea*	180
*Bombina sp.*	160
*Bufo sp.*	100
*Tylototriton taliangensis*	100
*Paramesotriton sp.*	100
*Leptobrachium sp.*	90
*Batrachuperus sp.*	76
*Salamandra salamandra*	20
*Rana chensinensis*	17
*Rana sp.*	10
*Brachytarsophrys carinensis*	4
	3601826

Amphibian trade information as recorded in the Law Enforcement Management Information System (LEMIS) maintained by the United States Fish and Wildlife Service (USFWS). Not all specimens were recorded at the species level upon importation; some only to genus and others as "Non-CITES amphibian species". Data is arranged in order of decreasing trade volume by recorded classification. Asterisk denotes species sampled in the current investigation; *Triturus hongkongensis* in not a currently recognized scientific name and is herein considered synonymous with *Paramesotriton hongkongensis*.

Amphibians were shipped communally in various quantities and arrived in bags of water with the exception of *Bombina orientalis*, which was shipped dry. In order to detect a minimum 10% prevalence within each shipment (with a 95% detection probability), we aimed to randomly sample 30 individuals of each species within each shipment [21]. Since this approach erroneously assumes both the sampling methodology and diagnostic test have 100% sensitivity, greater numbers were sampled when possible, although fewer were collected on occasion due to time constraints. Sampling effort was evenly distributed among all bags of amphibians within each shipment. All amphibians were randomly selected for sampling through blinding of the sampler, often from a bag containing hundreds of individuals, and sometimes included animals that were dead on arrival. Amphibians were temporarily housed in a separate container after processing and returned upon completion to prevent re-sampling of the same individuals.

Each shipment was unsealed and sampled immediately upon arrival in the USA, eliminating the risk of domestic or iatrogenic contamination. Fresh pairs of Nitrile gloves were worn for each shipment sampled. Amphibians were sampled for *Bd* using sterile fine-tipped rayon swabs with plastic shafts. The underside of the legs, feet and ventral surface were swabbed approximately five times each [22] and the swab bud was snapped off into a dry 2 mL cryovial. Samples were maintained dry at room temperature for a maximum of seven days before being transferred to a -80C freezer pending analysis.

Water samples from bags carrying amphibians were collected from each shipment and filtered to detect the presence of *Bd* following protocols established by Kirshtein et al. (2007) [23]. Immediately after a bag was opened and before *Bd* swabbing commenced, approximately 550 mL of water were extracted and sealed in a sterile container for subsequent filtration. This water was drawn into a sterile 60 mL syringe and pumped manually through a 0.22-micron Sterivex filter capsule until the filter became nearly clogged with organic debris. Then, 50 mL of phosphate buffered saline was passed through the capsule to rinse the filter before being pumped dry. After the addition of 0.9 mL Qiagen ATL lysis buffer with a sterile 1 mL syringe, the filter capsule was sealed and stored for subsequent qPCR analysis. Most species arrived in two separate bags of water (of which only one was sampled), except for the instances where a single shipment of *Xenopus laevis* arrived in four bags of water (of which two were sampled) and a single shipment of *B. orientalis* which arrived dry. A sealed bottle of spring water was filtered onsite to serve as a negative control to assess for equipment contamination.

Ranavirus sampling was performed by cloacal swabbing as described in Gray et al. (2012) [24]. Although this technique can underestimate the incidence of ranaviral infection by as much as 22% compared to lethal methods, only non-invasive sampling was allowed. All animals were first swabbed for *Bd* immediately upon removal from the container in which they arrived. Due to time constraints, only a subset of *Bd*-tested amphibians were subsequently sampled for ranavirus while still in hand. Swab buds were snapped off into a 2 mL cryovial containing 0.5 mL Nuclisens solution and stored under the same conditions as *Bd* samples while pending analysis. Due to the overlap in sample collection between ranavirus and *Bd*, all data collection parameters previously listed for *Bd* also apply to animals tested for ranavirus.

### Real-time PCR

Taqman PCR for *Bd* was generally based on the method, primers and probe of Boyle et al. (2003) [25]. For swab samples the DNA template was prepared with Prepman Ultra® (Applied Biosystems). Water filter samples were processed following the method of Kirshtein et al. (2007) [23]. Reactions used the Taqman Environmental Mastermix 2.0 (Applied Biosystems). Samples were run in triplicate on an ABI/Applied Biosystems 7900HT thermocycler using 384 well plates with an exogenous internal positive control labeled with VIC™ (Applied Biosystems) for each sample to detect PCR inhibitors. Samples that amplified at a Ct≥50 were considered negative. Samples amplifying at a Ct of <50 in 2 or more wells were considered positive. Those which produced a positive reaction in only one of three runs were considered "equivocal" and reported as negative in the data presented herein as recommended by Hyatt et al. (2007) [22] and Skerratt et al. (2011) [26] in order to maximize specificity. Quantification standards were created by growing *Bd* isolate JEL 197 on 1% tryptone agar and harvested of zoospores by rinsing plates with 1X PBS. After collection zoospores were counted three times on a hemocytometer to determine a range of zoospores ml ^−1^. Standard curves were generated with ten-fold serial dilutions (range 1×10^6^ to 1×10^−2^ zoospores). In addition to positive controls (quantification standards), each plate included a negative control (Taqman mastermix and no sample DNA) as well as 4 positive and negative quality assurance controls consisting of swabs either inoculated with *Bd* zoospores or sham-inoculated. The intensity of infection in positive samples was expressed as the number of zoospore equivalents per swab [27] or per liter of water.

Taqman PCR for ranavirus used primers, probes, and protocols as described by Pallister et al. (2007) [28], using the CON probe designed based on conserved segments of the ranavirus major capsid protein (MCP) gene. DNA was extracted from swabs using DNeasy Blood and Tissue Kits (QIAGEN Inc., Valencia, CA, USA) with spin columns, following the manufacturer’s protocol. The assay was performed using the ABI Real-time 7900HT system as described above. Samples amplifying at Ct’s of <50 in 3 or wells were considered positive. A standard curve was created by diluting a synthetic plasmid PIDTSMART-AMP (Integrated DNA Technologies, San Diego, CA) containing the ranavirus MCP gene primers and probe sequences for the conserved MCP gene region (Genbank 298256130) insert from the above. The plasmid was diluted in nuclease-free water from 10
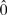
8 copies/5 ul in a series of eight 1∶10 dilutions down to 10 copies/5 ul and run in duplicate along with a third well containing the exogenous master mix (EIC, Life Technologies).

## Results

### Amphibian trade activity: exports from hong kong to the USA

Approximately 720,000 live amphibians were exported from Hong Kong and imported into the USA annually from 2006–2010 (Table 1). This activity involved no less than 31 species and consisted of those utilized primarily for the pet trade. Despite this diversity, few species composed the majority of traded specimens; those four sampled in this investigation collectively represented 40.9% of the 3.6 million amphibians supplied by Hong Kong during the 5-year period examined. Although exported amphibians were primarily documented to have been bred in captivity in Hong Kong, some were first imported from other Southeast Asian countries such as China, Indonesia, Singapore, and Thailand, and then re-exported from Hong Kong to the USA.

### Batrachochytrium dendrobatidis results

Five shipments of amphibians, representing four species and originating from two separate exporters in Hong Kong were examined for the presence of *Bd*. Both amphibians and water were tested from four shipments, but only water was tested in the fifth. Shipments were sampled at a single port of entry in the USA and arrived during a 19-week time period. Molecular analysis of skin swab samples by qPCR indicated the presence of *Bd* in 31/265 (11.7%) amphibians imported into the USA from Hong Kong, and in one of four shipments (Tables 2 & 3). Two of four species tested positive: *B. orientalis* and *X. laevis*. In the single shipment containing *Bd*-positive amphibians, the percentages of affected animals were 5.4% in *B. orientalis* and 70.0% in *X. laevis*. The average *Bd* zoospore equivalents per swab were consistently low, suggesting weak infection intensities.

**Table 2:** Cumulative *Bd* and *Ranavirus* detection in amphibians imported from Hong Kong.

Species	Common name	# *Bd*	*Bd+*	# RV	RV*+*	H_2_O *Bd*+	Sloughing	Ulcerations	DOA
*Bombina orientalis*	Oriental fire-bellied toad	56	3	13	10	-	22	0	3
*Cynops orientalis*	Oriental fire-bellied newt	97	0	78	60	-	7	4	15
*Paramesotriton hongkongensis*	Hong Kong newt	72	0	54	35	+	8	0	4
*Xenopus laevis*	African clawed frog	40	28	40	0	+	0	0	1
		265	31	185	105		37	4	23

Number of individuals sampled (#) for either *Bd* or ranavirus (RV), number of individuals testing positive by PCR (+), and presence of pathogen in water (H_2_O *Bd*+) are expressed. Animal condition recorded upon sampling is provided, including skin sloughing, ulcerations, and the number of sampled specimens that were dead on arrival (DOA).

**Table 3:** Presence of *Bd* and *Ranavirus* within individual amphibian shipments imported from Hong Kong.

Species	Shipment	Date of Import	Exporter	#/Shipment	# *Bd*	*Bd+*	# RV	RV*+*	H_2_O *Bd*+
*Cynops orientalis*	1	05/16/2012	A	500	36	0	35	35	N
*Paramesotriton hongkongensis*	1	05/16/2012	A	1600	36	0	36	35	Y
*Xenopus laevis*	2	06/06/2012	B	500	N/A	N/A	N/A	N/A	Y
*Cynops orientalis*	3	06/06/2012	A	500	36	0	18	0	N
*Paramesotriton hongkongensis*	3	06/06/2012	A	1600	36	0	18	0	Y
*Bombina orientalis*	4	09/26/2012	A	1000	56	3	13	10	N/A
*Xenopus laevis*	4	09/26/2012	A	1200	40	28	40	0	Y
*Cynops orientalis*	5	09/26/2012	B	200	25	0	25	25	N
				7100	265	31	185	105	

For each importation event, the number of animals present in the shipment, number of animals sampled (#*Bd*), number of those positive by PCR for infection (*Bd*+/RV+), and presence of pathogen in water (H_2_O *Bd*+) are expressed. The letter A or B reflects which exporter supplied the shipment. Note that in Shipment 2, only results for water filtration are available and not swab results for individual animals.

Filtration of water samples collected from five shipments of aquatic amphibians tested positive *Bd* via qPCR in 5/8 bags tested, demonstrating the presence of *Bd*-contaminated water in 4/5 shipments (Table 4). Each bag only contained members of a single species, and water holding two of the four species (*X. laevis* and *Paramesotriton hongkongensis*) tested positive. The same aquatic species (*X. laevis*) that tested positive by swabs also had water testing positive for *Bd*. In both shipments of *P. hongkongensis*, the water tested positive for *Bd* whereas all skin swabs tested negative. Water transporting *X. laevis* contained exceptionally high densities of *Bd*, and the average *Bd* zoospore equivalent per liter ranged from 3,390 to 16,887 in two shipments, whereas that for *P. hongkongensis* ranged from 2.9 to 5.7. Both exporters in Hong Kong supplied shipments containing *Bd*-contaminated water, whereas *Bd*-positive amphibians were only detected from one exporter. *Bd* was not detected in the water control sample processed onsite.

**Table 4:** Presence of *Bd* in water sampled from shipments of amphibians imported from Hong Kong.

Species	Shipment	Vol (mL)	ZSE
Cynops orientalis	1	360	ND
Paramesotriton hongkongensis	1	495	5.7
Xenopus laevis	2	515	6455
Cynops orientalis	3	480	ND
Paramesotriton hongkongensis	3	515	2.9
Xenopus laevis	4	325	3390
Xenopus laevis	4	310	16887
Cynops orientalis	5	125	ND

Volume of water processed is reflected in milliliters; *Bd* zoospore equivalents per liter (ZSE) represents the mean from three laboratory replicates; ND =  *Bd* not detected.

### Ranavirus results

Molecular analysis by qPCR indicated the presence of ranavirus in 105/185 (56.8%) of amphibians and in 3/4 shipments sampled (Tables 2 & 3). Individuals from all species tested positive for infection except *X. laevis*. Cumulative percentages of affected individuals were 76.9% in *B. orientalis,* 48.6% in *P. hongkongensis*, and 76.9% in *Cynops orientalis*. Although both ranavirus and *Bd* were concurrently detected in bags of amphibians in two shipments (Shipments 1 & 4; Table 3), co-infection was not observed in any individual animal sampled. Amphibians infected with ranavirus were detected in shipments from both Hong Kong exporters.

## Discussion

This investigation establishes the first record of *Bd* and *Ranavirus* presence in amphibian trade in Hong Kong and demonstrates an opportunity for exposure to native amphibians. In addition, this is the first report to the authors’ knowledge of ranavirus detection in the three species testing positive in this study. Unlike previous amphibian trade investigations in Southeast Asia [5], [29], a relatively high proportion of Hong Kong's traded animals tested positive for both of these significant pathogens. Risk of pathogen spillover and potential establishment is elevated by the regions' high volume of domestic trade in species with known pathogen susceptibility and the likelihood to persist in the wild in a wide range of habitats if released or escaped, including the Chinese bullfrog (*Hoplobatrachus rugulosus*) and African clawed frog (*X. laevis*) [4], [30], [31].

The previous lack of *Bd* detection in the wild and in Hong Kong trade by Rowley et al. (2007) [16] is surprising, given the findings of this study and the long-term presence of international amphibian trade in the region. Indications of amphibian escape or release from the exotic pet trade into the wild date back as far as 1977, with Japanese red-bellied newts (*Cynops pyrrhogaster*) recorded from Sha Tau Kok in the New Territories [32]. Due to the narrow diversity of native amphibian species previously evaluated for *Bd* infection and sampling bias towards post-metamorphic specimens, it remains possible that amphibian pathogens have evaded detection. Previous surveys throughout Asia have generally demonstrated widespread *Bd* distribution at low prevalence, and have provided some evidence for the presence of an endemic Asian lineage of *Bd* [5], [29], [31], [33]. Therefore, although the possibility of an historic introduction of *Bd* and long-term presence in Hong Kong cannot be fully disregarded, the absence of both prior detection and disease-suspected population declines suggests this phenomenon would be of low conservation concern relative to the contemporary importation of exotic disease strains that typically express higher virulence than strains considered endemic to the region [20], [34].

The risk of establishment of highly virulent trade-associated strains of *Bd* and ranavirus in Hong Kong depends largely on the continued importation and domestic sale of diseased amphibians. An analysis of trade activity from 2005–2006 showed the importation of nearly 4.3 million live amphibians into Hong Kong, comprised of at least 45 species originating from 11 countries [16], nine of which have reported presence of *Bd* and/or ranavirus in wild or traded herptiles [20], [35], [36]. The majority of this trade volume involved bullfrogs (*H. rugulosus*) intended for human consumption in Hong Kong and originated in either Thailand or China, where these pathogens have been detected in farmed and free-ranging amphibians [19], [15], [20], [37].

The risk of pathogen spillover from trade into the wild in Hong Kong is heightened by several factors additional to those mentioned above. First, each of these pathogens may cause mortality in the host with the absence of other clinical signs or lesions prior to death, which makes visual identification of illness difficult for traders. Only 21.3% of animals testing positive for *Bd* (1/31) or ranavirus (28/105) in this study were either dead on arrival (DOA) or had some form of visible lesion upon thorough inspection, demonstrating nearly 80% of animals carrying pathogens would have passed unnoticed. Specifically with respect to those DOA, most sampled for ranavirus were infected (15/19), whereas no DOA animals were positive for Bd (0/23). Furthermore, 23.5% of all animals testing negative for *Bd* (50/234) or *Ranavirus* (5/80) did display lesions or were DOA. Therefore, identification of potential signs of illness or disease does not accurately represent risk from these pathogens and cannot be used as an effective means for traders to exclude infected animals from commerce.

Second, infectious ranavirus and *Bd* particles released by affected animals into their environment can survive for extended periods of time outside the amphibian host; ranging from at least three to seven weeks respectively, and potentially longer under optimal conditions [8], [38]. This prolonged persistence extends the window of opportunity for native amphibians to become exposed to infectious particles if untreated disposal of contaminated water were to occur. For this reason, the World Organization for Animal Health (OIE) suggests the disinfection of any water, containers, or other surfaces that had contact with amphibians prior to disposal [39]. As no such measures are enforced in Hong Kong, disease outbreaks may occur even without the escape or release of infected animals. It is unknown how often facilities in Hong Kong are disposing pathogen-contaminated water in a manner exposing local wildlife, but Gilbert et al. (2013) [5] found disposal directly into the environment to be common practice among all surveyed frog-farming facilities in Vietnam. Consequently, the abundance of aquatic amphibian species traded by Hong Kong (Table 1), prolonged environmental persistence of infectious ranavirus and *Bd* particles, and employment of trade activities that neither disinfect water nor safely dispose of deceased animals creates an ideal pathway for disease transmission to native Hong Kong amphibians.

The practice of disease surveillance within the international wildlife trade is a relatively low-cost and rapid technique to detect pathogen presence in any given country of interest that engages in trade of a potential host species. Once identified, opportunities for pathogen spillover from the trade sector and subsequent exposure to native species can be investigated and managed to control spread and prevent a potential outbreak. Although the data produced remains specific to that of traded amphibians and cannot be used to draw inferences about disease status in wild amphibians, results from trade surveys can provide invaluable information about the physical presence of a pathogen in a region of uncertain status before detection in wild populations, as we have demonstrated. If soon detected in native amphibians, it will be important to discern the presence of endemic pathogen strains from those introduced by traded amphibians and remain particularly vigilant if foreign sources are suspected, as both ranavirus and *Bd* associated with commercial trade often express greater virulence [20], [34].

Data produced by this investigation provides guidance for the design of surveys to determine the pathogen status of future amphibian shipments. The consistently high prevalence of ranavirus detected by cloacal swabbing suggests that relatively little sampling effort was required to identify its presence in all affected species, whereas a smaller number of skin swabs for *Bd* detection may have resulted in the false-negative classification of *B. orientalis*. Filtration of the water carrying amphibians consistently provided greater sensitivity for the detection of *Bd* than skin swabs (i.e. detection in 3/4 shipments via filtering versus 1/4 via swabs), likely due to the collective sampling of *Bd* zoospores from a larger pool of animals than those individually sampled. It is important to note the possibility that the *P. hongkongensis* tested in this study may have been shipped in *Bd*-contaminated water and not themselves been infected, but this detail is irrelevant where primary survey intent is to detect pathogen presence in a shipment rather than prevalence. Still, it is surprising that all 72 *P. hongkongensis* tested negative for *Bd*, despite their immersion in Bd-positive water and the added potential for contamination caused by the water residue on each animal sampled. In summation, these data suggest an efficient screening method to identify pathogen presence in high volume aquatic amphibian shipments needs only to focus swabbing efforts on ranavirus detection and filter a sample of water to detect *Bd,* if knowledge of prevalence is not required.

We have demonstrated the presence of *Bd* and ranavirus in Hong Kong's trade sector and show that the risk of spillover through contaminated wastewater is particularly high. Considering these and prior findings, a limited window of opportunity exists to protect the region's 24 species of native amphibians from trade-associated pathogen exposure and potential decline. Eradication of these pathogens from wild amphibian populations is not known to be possible following establishment, calling for greater vigilance and proactive surveillance in high-risk regions where they have yet to be detected. Control over the presence of ranavirus and *Bd* in Hong Kong, a major hub of international amphibian trade, would likewise benefit global efforts to reduce the dispersal of these devastating amphibian pathogens.

## References

[1] Fisher MC, Garner TWJ (2007) The relationship between the emergence of *Batrachochytrium dendrobatidis*, the international trade in amphibians and introduced amphibian species. Fungal Biology Reviews 21: 2–9.

[2] Schloegel LM, Picco A, Kilpatrick AM, Hyatt A, Daszak P (2009) Magnitude of the US trade in amphibians and presence of *Batrachochytrium dendrobatidis* and ranavirus infection in imported North American bullfrogs (*Rana catesbeiana*). Biological Conservation 142: 1420–1426.

[3] Schloegel LM, Toledo LF, Longcore JE, Greenspan SE, Vieira CA, et al (2012) Novel, panzootic and hybrid genotypes of amphibian chytridiomycosis associated with the bullfrog trade. Molecular Ecology 21(21): 5162–5177. 22857789 10.1111/j.1365-294X.2012.05710.x

[4] Weldon C, du Preez LH, Hyatt AD, Muller R, Speare R (2004) Origin of the amphibian chytrid fungus. Emerging Infectious Diseases 10: 2100–2105. 15663845 10.3201/eid1012.030804 PMC3323396

[5] Gilbert M, Bickford D, Clark L, Johnson A, Joyner PH, et al (2013) Amphibian pathogens in Southeast Asian frog trade. Ecohealth 9(4): 386–98. 10.1007/s10393-013-0817-7 23404036

[6] Daszak P, Strieby A, Cunningham AA, Longcore JE, Brown CC, et al (2004) *Experimental evidence that the bullfrog (Rana catesbeiana*) is a potential carrier of chytridiomycosis, an emerging fungal disease of amphibians. Herpetological Journal 14(4): 201–207.

[7] Hanselmann R, Rodriguez A, Lampo M, Fajardo-Ramos L, Aguirre AA, et al (2004) Presence of an emerging pathogen of amphibians in introduced bullfrogs *Rana catesbiana* in Venezuela. Biological Conservation 120: 115–119.

[8] Johnson M, Speare R (2003) Survival of *Batrachochytrium dendrobatidi*s in water: quarantine and control implications. Emerging Infectious Diseases 9(8): 922–925. 12967488 10.3201/eid0908.030145 PMC3020615

[9] Johnson M, Speare R (2005) Possible modes of dissemination of the amphibian chytrid *Batrachochytrium dendrobatidis* in the environment. Diseases of Aquatic Organisms 65: 181–186. 16119886 10.3354/dao065181

[10] Teacher AGF, Cunningham AA, Garner TWJ (2010) Assessing the long-term impact of ranavirus infections on wild common frog (*Rana temporaria*) populations. Animal Conservation 13: 514 – 522.

[11] Gray MJ, Miller DL, Hoverman JT (2009) Ecology and pathology of amphibian ranaviruses. Diseases of Aquatic Organisms 87: 243–266. 20099417 10.3354/dao02138

[12] Picco AM, Collins JP (2008) Amphibian commerce as a likely source of pathogen pollution. Conservation Biology 22: 1582–1589. 18717688 10.1111/j.1523-1739.2008.01025.x

[13] Une Y, Sakuma A, Matsueda H, Nakai K, Murakami M (2009) Ranavirus outbreak in North American bullfrogs (*Rana catesbeiana*), Japan, 2008 [letter]. Emerging Infectious Diseases 15(7): 1146–1147. 19624949 10.3201/eid1507.081636 PMC2744262

[14] Xu K, Zhu DZ, Wei Y, Schloegel LM, Chen XF, et al (2010) Broad distribution of ranavirus in free-ranging *Rana dybowskii* in Heilongjiang, China. EcoHealth 7(1): 18–23. 20217181 10.1007/s10393-010-0289-y

[15] Vörös J, Satasook C, Bates P, Wangkulangkul S (2012) First record of the amphibian chytrid fungus, *Batrachochytrium dendrobatidis* in Thailand. Herpetology Notes 5: 519–521.

[16] Rowley JJL, Chan SKF, Tang WS, Speare R, Skerratt LF, et al (2007) Survey for the amphibian chytrid *Batrachochytrium dendrobatidis* in Hong Kong in native amphibians and in the international amphibian trade. Diseases of Aquatic Organisms 78: 87–95. 18286805 10.3354/dao01861

[17] Kolby JE, Padgett-Floher GE, Field R (2010) Amphibian Chytrid Fungus *Batrachochytrium dendrobatidis* in Cusuco National Park, Honduras. Diseases of Aquatic Organisms 92: 245–51. 21268988 10.3354/dao02055

[18] Skerratt LF, Berger L, Hines HB, McDonald KR, Mendez D, et al (2008) Survey protocol for detecting chytridiomycosis in all Australian frog populations. Diseases of Aquatic Organisms 80(2): 85–94. 18717061 10.3354/dao01923

[19] Skerratt LF, Phillott AD, Cashins SD, Webb R, Puschendorf R, et al.. (2010) Experimental Research to Obtain a Better Understanding of the Epidemiology, Transmission and Dispersal of Amphibian Chytrid Fungus in Australian Ecosystems. Report. Australian Government Department of Environment and Heritage.

[20] Miller DL, Gray MJ, Storfer A (2011) Ecopathology of ranaviruses infecting amphibians. Viruses 3: 2351–2373. 22163349 10.3390/v3112351 PMC3230856

[21] Thrusfield M (2005) Veterinary Epidemiology, 3rd edition. Blackwell Science Ltd.

[22] Hyatt AD, Boyle DG, Olsen V, Boyle DB, Berger L, et al (2007) Diagnostic assays and sampling protocols for the detection of *Batrachochytrium dendrobatidis* . Diseases of Aquatic Organisms 73: 175–192. 17330737 10.3354/dao073175

[23] Kirshtein JD, Anderson CW, Wood JS, Longcore JE, Voytek MA (2007) Quantitative PCR detection of *Batrachochytrium dendrobatidis* DNA from sediments and water. Diseases of Aquatic Organisms 77: 11–15. 17933393 10.3354/dao01831

[24] Gray MJ, Miller DL, Hoverman JT (2012) Reliability of non-lethal surveillance methods for detecting ranavirus infection. Diseases of Aquatic Organisms 99: 1–6. 22585297 10.3354/dao02436

[25] Boyle DG, Boyle DB, Olsen V, Morgan JAT, Hyatt AD (2004) Rapid quantitative detection of chytridiomycosis (*Batrachochytrium dendrobatidis*) in amphibian samples using real-time Taqman PCR assay. Diseases of Aquatic Organisms 60: 141–148. 15460858 10.3354/dao060141

[26] Skerratt LF, Mendez D, McDonald KR, Garland S, Livingstone J, et al (2011) Validation of diagnostic tests in wildlife: the case of chytridiomycosis in wild amphibians. Journal of Herpetology 45(4): 444–450.

[27] Vredenburg VT, Knapp RA, Tunstall T, Briggs CJ (2010) Large-scale Amphibian Die-offs Driven by the Dynamics of an Emerging Infectious Disease. Proceedings of the National Academy of Sciences of the Unites States of Amercia 107(21): 9689–9694. 10.1073/pnas.0914111107 PMC2906868 20457913

[28] Pallister J, Gould A, Harrison D, Hyatt A, Jancovich J, et al (2007) Development of real-time PCR assays for the detection and differentiation of Australian and European ranaviruses. Journal of Fish Diseases 30: 427–438. 17584440 10.1111/j.1365-2761.2007.00828.x

[29] Swei A, Rowley JJL, Rödder D, Diesmos MLL, Diesmos AC, et al (2011) Is Chytridiomycosis an Emerging Infectious Disease in Asia? PLoS ONE 6(8): e23179. 21887238 10.1371/journal.pone.0023179 PMC3156717

[30] Rowley J, Brown R, Kusrini M, Inger R, Stuart B, et al (2010) Impending conservation crisis for Southeast Asian amphibians. Biology Letters 6: 336–338. 20007165 10.1098/rsbl.2009.0793 PMC2880038

[31] Bai C, Garner TWJ, Li Y (2010) First evidence of *Batrachochytrium dendrobatidis* in China: discovery of chytridiomycosis in introduced American bullfrogs and native amphibians in the Yunnan Province, China. EcoHealth 7(1): 127–134. 20372969 10.1007/s10393-010-0307-0

[32] Risch J, Romer JD (1980) Origin and taxonomic status of the salamander *Cynops shataukokensis* . Journal of Herpetology 14: 337–341.

[33] Bai C, Liu X, Fisher MC, Garner TWJ, Li Y (2012) Global and endemic Asian lineages of the emerging pathogenic fungus *Batrachochytrium dendrobatidis* widely infect amphibians in China. Diversity and Distributions 18: 307–318.

[34] Farrer RA, Weinert LA, Bielby J, Garner TW, Balloux F, et al (2011) Multiple emergences of genetically diverse amphibian-infecting chytrids include a globalized hypervirulent recombinant lineage. Proceedings of the National Academy of Sciences of the United States of America 108(46): 18732–6. 22065772 10.1073/pnas.1111915108 PMC3219125

[35] Stöhr AC, Hoffmann A, Papp T, Robert N, Pruvost NBM, et al (2013) Long-term study of an infection with ranaviruses in a group of edible frogs (*Pelophylax kl. esculentus*) and partial characterization of two viruses based on four genomic regions. The Veterinary Journal 197(2): 238–244. 23535222 10.1016/j.tvjl.2013.02.014

[36] Olson DH, Aanensen DM, Ronnenberg KL, Powell CI, Walker SF, et al (2013) Mapping the Global Emergence of *Batrachochytrium dendrobatidis*, the Amphibian Chytrid Fungus. PLoS ONE 8(2): e56802 doi:10.1371/journal.pone.0056802 23463502 10.1371/journal.pone.0056802 PMC3584086

[37] Geng Y, Wang KY, Zhou ZY, Li CW, Wang J, et al (2011) First report of a Ranavirus associated with morbidity and mortality in farmed Chinese Giant Salamanders (*Andrias davidianus*). Journal of Comparative Pathology 145: 95–102. 21256507 10.1016/j.jcpa.2010.11.012

[38] Nazir J, Spengler M, Marschang RE (2012) Environmental persistence of amphibian and reptilian ranaviruses. Diseases of Aquatic Organisms 98: 177–184. 22535867 10.3354/dao02443

[39] Schloegel LM, Daszak P, Cunningham AA, Speare R, Hill B (2010) Two amphibian diseases, chytridiomycosis and ranaviral disease, are now globally notifiable to the World Organization for Animal Health (OIE): an assessment. Diseases of Aquatic Organisms 92: 101–108. 21268971 10.3354/dao02140

